# Comparison of Different Liquid Chromatography-Based Purification Strategies for Adeno-Associated Virus Vectors

**DOI:** 10.3390/pharmaceutics13050748

**Published:** 2021-05-18

**Authors:** Ruth Rieser, Johanna Koch, Greta Faccioli, Klaus Richter, Tim Menzen, Martin Biel, Gerhard Winter, Stylianos Michalakis

**Affiliations:** 1 Department of Pharmacy—Center for Drug Research, Ludwig-Maximilians-Universität München, Butenandtstr. 5-13, 81377 Munich, Germany; ruth.rieser@cup.uni-muenchen.de (R.R.); johanna.Koch@cup.uni-muenchen.de (J.K.); greta.faccioli3@gmail.com (G.F.); martin.biel@cup.uni-muenchen.de (M.B.); 2 Coriolis Pharma, Fraunhoferstr. 18 b, 82152 Martinsried, Germany; klaus.richter@coriolis-pharma.com (K.R.); tim.menzen@coriolis-pharma.com (T.M.); 3 Department of Ophthalmology, University Hospital, LMU Munich, Mathildenstr. 8, 80336 Munich, Germany

**Keywords:** adeno-associated virus, rAAV vector, column purification, empty capsids, ion exchange chromatography, affinity chromatography

## Abstract

Recombinant adeno-associated virus (rAAV) vectors have evolved as one of the most promising technologies for gene therapy due to their good safety profile, high transduction efficacy, and long-term gene expression in nondividing cells. rAAV-based gene therapy holds great promise for treating genetic disorders like inherited blindness, muscular atrophy, or bleeding disorders. There is a high demand for efficient and scalable production and purification methods for rAAVs. This is particularly true for the downstream purification methods. The current standard methods are based on multiple steps of gradient ultracentrifugation, which allow for the purification and enrichment of full rAAV particles, but the scale up of this method is challenging. Here, we explored fast, scalable, and universal liquid chromatography-based strategies for the purification of rAAVs. In contrast to the hydrophobic interaction chromatography (HIC), where a substantial amount of AAV was lost, the cation exchange chromatography (CEX) was performed robustly for multiple tested serotypes and resulted in a mixture of full and empty rAAVs with a good purity profile. For the used affinity chromatography (AC), a serotype dependence was observed. Anion exchange chromatography (AEX) worked well for the AAV8 serotype and achieved high levels of purification and a baseline separation of full and empty rAAVs. Depending on the AAV serotype, a combination of CEX and AEX or AC and AEX is recommended and holds promise for future translational projects that require highly pure and full particle-enriched rAAVs.

## 1. Introduction

Recombinant adeno-associated viral (rAAV) vectors have emerged as very promising gene delivery tools and have been already used in more than 190 clinical trials across different indications with very good safety characteristics [1,2,3,4]. A high number of immunologically distinct serotypes of AAV are currently in use; however, rAAV based on serotype 2 (AAV 2) have been most extensively evaluated in preclinical studies, as this was the first serotype to be fully characterized [5]. Accordingly, there is a growing need for scalable commercial production and purification methods. The manufacturing processes for rAAVs are composed of three phases: (i) upstream, which entails the production of the rAAVs, (ii) downstream, which involves the purification of the AAVs, and (iii) formulation/fill and finish processes, which ensure optimal stability and dosing at the desired therapeutic dose [6]. Here, we focus on the downstream purification process.

In the past two decades, many protocols for the purification of rAAV vectors have been established [7,8,9,10,11,12,13,14]. Downstream purification processes are designed to remove process-related impurities such as host cell proteins, host cell DNA, and process additives, as well as product-related impurities, such as aggregated vectors and empty capsids that are generated during the upstream process [15]. One way to purify rAAV vectors is cesium chloride (CsCl) or iodixanol gradient ultracentrifugation, and the material purified by such methods has been used in several clinical trials [16,17,18]. The main advantage of these gradient ultracentrifugation protocols is the flexibility to be used with minimal adaptation to efficiently purify different AAV serotypes and the ability to separate full rAAVs from empty particles. Nevertheless, gradient ultracentrifugation methods are typically limited to product volumes of a few hundred microliters, are not readily scalable, and are difficult to run under good manufacturing practice (GMP). In contrast, the liquid chromatography-based purification methods offer scalable alternative methods that enable the manufacturing of rAAVs with high purity, potency, and consistency. However, chromatography resins and conditions are often uniquely developed and tailored for each serotype, and the efficient removal of vector-related impurities such as empty capsids is challenging [8]. The impact of empty capsids on the potency and immunogenicity is discussed [19], and therefore, protocols for their efficient removal are desired and currently explored by several groups and companies. Here, we present data on the liquid chromatography column-based purification processes for AAV2/8, comparing affinity chromatography (AC) with hydrophobic interaction chromatography (HIC) and cation exchange chromatography (CEX), followed, in both cases, by anion exchange chromatography (AEX) to separate full from empty capsids. In AEX method development, we were able to achieve a baseline separation between genome-loaded and empty capsids.

The protocol of the AC and CEX could also be applied for the purification of AAV2 and resulted in a similar level of purification.

## 2. Materials and Methods

### 2.1. AAV Production

Recombinant AAV vectors with genomes carrying inverted terminal repeats derived from AAV2 and packaged with wildtype AAV2 or AAV8 or engineered AAV2 capsids (AAV2.NN and AAV2.GL) were produced and purified as previously described in adherent HEK293T cells (The Leibniz Institute DSMZ-German Collection of Microorganisms and Cell Cultures GmbH, Braunschweig, Germany) [20,21]. The packaged self-complementary (sc) genome comprised a CMV promoter driving expression of eGFP [22].

### 2.2. Tangential Flow Filtration—TFF

For some rAAV vector batches, the cell culture supernatant was harvested, filtered, and concentrated by tangential flow filtration (TFF) performed with a KrosFlo^®^ KRi2 universal using a 100-kDa mPES membrane from Repligen (Waltham, MA, USA) and buffered in 1× PBS (10-mM Na_2_HPO_4_, 1.8-mM KH_2_PO_4_, 4.27-mM KCl, and 137-mM NaCl, pH 7.4).

### 2.3. PEG Precipitation

For other rAAV vector batches, the cell culture supernatant was harvested three days and, for AAV8, also six days after transfection and filtered with a 0.45-µm filter. Subsequently, polyethylene glycol (PEG) 8000 (Merck, Darmstadt, Germany) was added to the supernatant to a final concentration of 8% (*v/v*). The mixture was centrifuged for 15 min at 1756× *g* at 4 °C. The supernatant was discarded, and the pellet was resuspended with 7.5 mL of PBS. To degrade free DNA, 2-µL Benzonase (Merck, Darmstadt, Germany; final concentration 50 U/mL) was added to the sample and incubated at 37 °C for 30 min and subsequently purified on the affinity column (AC), hydrophobic interaction chromatography (HIC) column, or directly by the cation exchange column (CEX).

### 2.4. Cell Pellet

In case the rAAV vectors were to be harvested from the cell pellet, the cells were detached from the culture dishes with a cell scraper, for AAV2 three and for AAV8 six days after transfection, collected into a centrifuge beaker, and pelleted by centrifugation at 1756× *g* at 4 °C for 15 min. Subsequently, the supernatant was decanted, and the cell pellet was resuspended in 7.5 mL of 50-mM Tris(hydroxymethyl)aminomethane (Tris)-HCl and 150-mM NaCl, pH 8.5. The resuspended cells were frozen three times with liquid nitrogen and thawed to 37 °C. After the last cycle of thawing, 2 µL of Benzonase (Merck, Darmstadt, Germany; final concentration 50 U/mL) were added and incubated for 30 min at 37 °C, followed by centrifugation at 4 °C, 1756× *g* for 25 min to separate the pellet and supernatant. The supernatant was collected and subsequently purified on the affinity column (AC), hydrophobic interaction chromatography (HIC) column, or to the cation exchange column (CEX).

### 2.5. rAAV Vector Purification

All purifications using liquid chromatography were performed with an ÄKTA purifier from Cytiva (Marlborough, MA, USA). For sample loading, a Superloop or sample pump were used. All runs were performed at room temperature (25 °C), and the detection was done with a UV detector (UL-9, fixed wavelength) at 280 nm implemented in the ÄKTA system. The evaluation was done with Unicorn Software (7.3) (Cytiva, Marlborough, MA, USA).

#### 2.5.1. Hydrophobic Interaction Chromatography—HIC

The CIMmultus OH monolith column (1 mL, 2-µm pores; BIA separations, Ajdovščina, Slovenia) was used for HIC. The sample was diluted 2-fold with 3-M K_2_HPO_4_ (Merck, Darmstadt, Germany), 2% glucose (Sigma-Aldrich Chemie GmbH, Steinheim, Germany), pH 7.0, and then filtrated over a 0.45-µm mPES membrane filter. After equilibration with 1.5-M K_2_HPO_4_ and 1% glucose, pH 7.0, the sample was loaded onto the column with a flow rate of 2 mL/min. For elution, the flow rate was set to 3 mL/min, and the gradient was run in 20 column volumes (CV) from 0% to 100% 50-mM K_2_HPO_4_ and 1% glucose, pH 7.0. The columns were washed in the cleaning in place (CIP) mode with 1-M NaOH (VWR, Geldenaaksenbaan, Belgium) for reuse [23].

#### 2.5.2. Cation Exchange Chromatography—CEX

The CIMmultus SO_3_ column (1 mL, 2-µm pores; BIA separations) was used for CEX chromatography. The pooled peak fractions from the HIC, the PEG precipitates, or the cell lysates were diluted 10-fold with 25-mM sodium acetate (C_2_H_3_NaO_2_) (Grüssing, Filsum, Germany) and 50-mM sodium chloride (NaCl) (Bernd Kraft, Duisburg, Germany) to achieve a conductivity of 15 to 20 mS/cm. The pH was adjusted to 4.0. After column equilibration with 50-mM sodium acetate and 100-mM NaCl, pH 4.0, the loading of the diluted sample was done at a flow rate of 5 mL/min. Elution was run with a 3-mL/min flow rate and a gradient ranging from 0% to 100% 50-mM C_2_H_3_NaO_2_ and 2-M NaCl, pH 4.0, within 20 CVs. The column was washed in the cleaning in place (CIP) mode with 1-M NaOH and 2-M NaCl after each run.

#### 2.5.3. Anion Exchange Chromatography—AEX—Salt Gradient

The final purification step to separate the full and empty rAAV particles was performed with the CIMmultus QA column (1 mL, 2-µm pores; BIA separations). The peak containing fractions from the CEX or AC elutions were pooled and diluted 30-fold with 20-mM Tris (Sigma-Aldrich Chemie GmbH, Steinheim, Germany) and 2-mM MgCl_2_ (Applichem GmbH, Darmstadt, Germany) and adjusted to pH 9.0. Required starting conductivity for the AEX was around 3–5 mS/cm. After column equilibration with 20-mM Tris and 2-mM MgCl_2_, pH 9.0, the loading of the diluted sample was done at a flow rate of 6 mL/min. Elution was performed at a 3-mL/min flow and a gradient ranging from 5% to 50% 20-mM Tris, 2-mM MgCl_2_, and 500-mM NaCl, pH 9.0, within 60 CVs. The column was cleaned in the cleaning in place (CIP) mode with 1-M NaOH and 2-M NaCl after each run [23,24].

#### 2.5.4. Anion Exchange Chromatography—AEX—pH Gradient

The CIMmultus PrimaS (AAV) column (1 mL, 2-µm pores; BIA separations) was evaluated as an additional option for separating empty from full rAAVs. Buffer A contained 10-mM Tris, 10-mM bis-tris-propane (Sigma-Aldrich Chemie GmbH, Steinheim, Germany), 2-mM MgCl_2_, and 0.1% poloxamer 188 (Kolliphor P 188, BASF, Ludwigshafen, Germany) at pH 8.0, and buffer B had the same composition but at pH 10.0. To achieve a conductivity below 4 mS/cm, the samples from CEX or AC were diluted 30-fold with dilution buffer (buffer A diluted 2-fold with water), and the pH was adjusted to 8.0. Loading and elution were performed at a flow rate of 4 mL/min and a linear pH gradient ranging from 0% to 100% B within 100 CVs. In some cases, a step gradient was used.

#### 2.5.5. Affinity Chromatography—AC

For affinity chromatography (AC), a Poros capture select AAVx column (1 mL; Thermo Fisher Scientific, Dreieich, Germany) was used. AAV samples in PBS at pH 7.4 were loaded at a flow rate of 0.3 mL/min on the column and eluted with 100-mM citric acid (VWR, Geldenaaksenbaan, Belgium), pH 3.0, at a flow rate of 0.7 mL/min. AAVs were neutralized by elution into 1-M Tris, pH 8.7. The used column was purified by cleaning in place with 100-mM phosphoric acid (Merck, Darmstadt, Germany), pH 2.0, and 6-M guanidine HCl (Sigma-Aldrich Chemie GmbH, Steinheim, Germany).

### 2.6. Vector Genome Titer Determination by qPCR

The vector titer was measured by real-time quantitative polymerase chain reaction (qPCR) and carried out with the QuantStudio 5 system (Thermo Fisher scientific, Dreieich, Germany). AAV2-free ITR qPCR was performed as described by D’costa et al. [25].

### 2.7. Determination of HEK 293T DNA

Determination of HEK cell DNA was done on a QuantStudio 5 system (Thermo Fisher Scientific, Dreieich, Germany) with a SYBR Green qPCR assay designed to amplify a 94-bp Alu sequence using the following primers: 5′-GAGGCGGGCGGATCA-3′ (forward) and 5′-CCCGGCTAATTTTTGTATTTTTAG-3′ (reverse) [26].

### 2.8. Sodium Dodecyl Sulfate Polyacrylamide Gel Electrophoresis—SDS-PAGE

Samples were loaded on a 6–12% Tris-HCl gel after the addition of Laemmli 4× (Bio-Rad, Hercules, CA, USA) and denaturation at 95 °C for 5 min. After electrophoresis for 40 min at 200 V in denaturing conditions, the gel was silver-stained and pictures were taken by ChemiDog (Bio-Rad, Feldkirchen, Germany) [27].

### 2.9. Sedimentation Velocity Analytical Ultracentrifugation—SV-AUC

Sedimentation velocity analytical ultracentrifugation (SV-AUC) was performed on an Optima AUC instrument (Beckman Coulter, Brea, CA, USA). rAAV samples were loaded into AUC cells with standard 2-sector centerpieces (Beckman Coulter, Brea, CA, USA) into both sectors. Sedimentation of particles was monitored at 16,336× *g* at the wavelengths of 230 nm, 260 nm, and 280 nm in an AN Ti-50 rotor (Beckman Coulter, Brea, CA, USA). Data analysis was then performed in UltraScan III (AUC Solutions, Katy, TX, USA) using the intensity signal of each sample cell. Data were processed to contain the relevant scan range and optimal solutions for the time-invariant and radial-invariant noises, which were determined together with the optimal meniscus position. Final fitting was performed with the PCSA-SL-MC model and the statistical evaluation based on 100 Monte-Carlo simulations. Peak integration was performed to derive the relative contents of the empty (~65 S) and filled capsids (~105 S) and of the formulation components, such as small proteins, DNA fragments, and smaller unidentified species. For determination of the full/empty ratio, the same factors for 260 nm and 280 nm were used as in the analytical AEX experiments, and for 230 nm, a correction factor of 1.6 was used.

### 2.10. Analytical Anion Exchange Chromatography

For the analytical separation of full and empty rAAVs, a Waters 2695 system (Eschborn, Germany) and a Protein-Pak Hi Res Q column (5 µm, 4.6 × 100 mm; Waters GmbH, Eschborn, Germany) were used. Therefore, 70-mM bis-tris-propane (Sigma-Aldrich, St. Louis, MO, USA) and 2-mM MgCl_2_, pH 9.0, were used as buffer A. Adding 1-M tetramethylammoniumchlorid (Sigma-Aldrich, St. Louis, MO, USA) to buffer A created buffer B. At a flow rate of 0.3 mL/min, a gradient from 10% to 30% B in 20 min was performed to separate the full and empty AAVs. rAAV elution was detected at 280 nm and 260 nm with a UV detector (Waters 2487) and with a fluorescence detector (Dionex RF2000, Sunnyvale, CA, USA) at excitation and emission wavelengths of 280 nm 330 nm, respectively. The chromatograms were integrated with Chromeleon V6.8 (Thermo Fisher Scientific, Dreieich, Germany). For the correct determination of a fraction of empty rAAVs, the following equation, according to Reference [28], was used (Equation (1)):

(1) $$\%\;Empty\;capsids=\;100\;\times \;\frac{Area_{empty}}{\left(Area_{empty}+\frac{Area_{full}}{RF_{F/E}}\right)}$$

*RF_F/E_* fluorescence detection: 0.85, UV 260 nm: 7.69, and UV 280 nm: 3.71.

## 3. Results

### 3.1. Evaluation of Two Different Liquid Chromatography-Based Purification Stragies for rAAV2/8 Vectors

**Figure 1:**
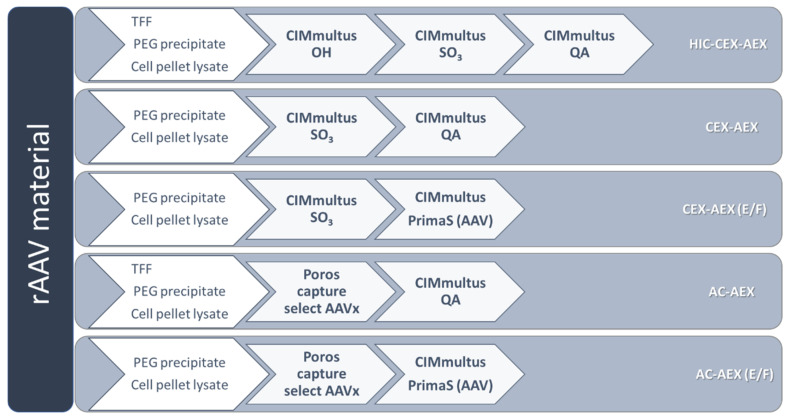
Overview of the five tested purification strategies to purify and to separate the empty and full recombinant adeno-associated virus (rAAV). For each experiment, only one harvesting method mentioned in the first arrow was performed. The abbreviations (in white) on the right side are also used throughout the text and refer here to the shown purification processes. AC, affinity chromatography; AEX, anion exchange chromatography; CEX, cation exchange chromatography; HIC, hydrophobic interaction chromatography; PEG, polyethylene glycol; TFF, tangential flow filtration.

**Figure 2:**
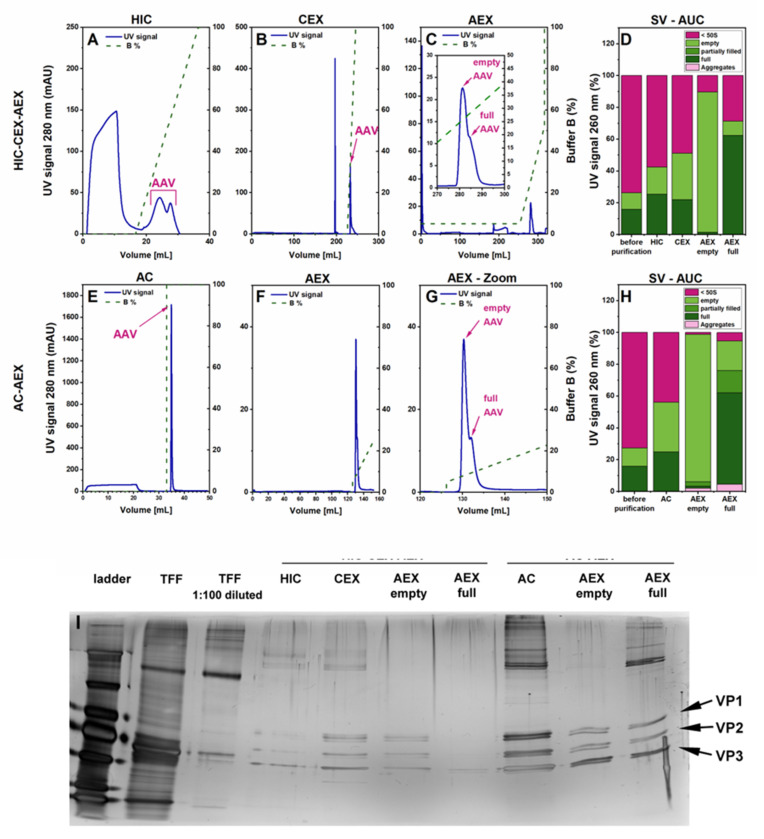
Comparison of HIC-CEX-AEX and AC-AEX for purification of the rAAV2/8 vectors. Each chromatogram shows the respective ultra-violet (UV) signal in blue (scale on the left *Y*-axis) and the concentration of buffer B depicted as a dashed green line (scale on the right). (**A**–**C**) Chromatograms obtained with HIC-CEX-AEX in three subsequent steps on a HIC (**A**), a CEX (**B**), and an AEX (**C**) column. (**D**) SV-AUC analysis of rAAV2/8 of the indicated samples from different purification states. (**E**,**F**) Chromatograms obtained with AC-AEX in two subsequent steps on an AC (**E**) and an AEX (**F**) column. (**G**) Magnification view of the AEX chromatogram shown in F (focusing on the 120–150-mL range) to better visualize the peaks of the empty and full rAAVs. (**H**) SV-AUC analysis after AC and AEX. (**I**) Silver-stained sodium dodecyl sulfate polyacrylamide gel electrophoresis (SDS-PAGE) of the rAAV material after TFF, and the intermediate steps from the purification downstream processes HIC-CEX-AEX and AC-AEX. AC, affinity chromatography; AEX, anion exchange chromatography; CEX, cation exchange chromatography; HIC, hydrophobic interaction chromatography; PEG, polyethylene glycol; rAAV, recombinant adeno-associated virus; TFF, tangential flow filtration; VP, viral protein.

We evaluated different purification strategies based on the liquid chromatography columns (Figure 1). For the initial testing, we decided to work with AAV8-pseudotyped AAV2 (rAAV2/8) vectors carrying a self-complementary (sc) genome with a CMV_eGFP gene expression cassette. The harvested rAAV vectors from the same batch were split into two parts, which were subsequently purified with different purification strategies. Purification process HIC-CEX-AEX was a three-step process consisting of hydrophobic interaction chromatography (HIC), followed by cation exchange chromatography (CEX) and, finally, anion exchange chromatography (AEX). Figure 2 summarizes the results obtained with HIC-CEX-AEX and AC-AEX for rAAV2/8. In the HIC-CEX-AEX process, the combination of HIC and CEX successfully removed most of the DNA and protein impurities (Figure 2A,B), and the final AEX step was able to separate, at least to some extent, the full and empty rAAVs (Figure 2C).

In contrast, AC-AEX is a two-step process and starts with affinity chromatography (AC) on a Poros capture select AAVx column, which can selectively capture AAV capsids [10,29,30,31], while most of the DNA and protein impurities are not retained on the column (Figure 2E). In the second step, the same AEX as in HIC-CEX-AEX was used (Figure 2F,G).

To better characterize the purification process, we compared the eluates containing rAAVs from each purification step with the starting material using sedimentation velocity analytical ultracentrifugation (SV-AUC). The SV-AUC results for HIC-CEX-AEX and AC-AEX are depicted in Figure 2D,H, respectively. In both cases, a relative increase of the rAAV-containing fractions was observed after each purification step, indicative of an improvement in the purity. A separation of full rAAVs from empty capsids could also be observed with HIC-CEX-AEX and AC-AEX. The highest purity was achieved for the empty capsid fraction, which seemed to be devoid of impurities and full capsids. While the full capsid fraction showed a clear enrichment, this fraction in HIC-CEX-AEX and AC-AEX still contained some empty capsids and other impurities (Figure 2D,H). Some degree of aggregation was observed after the AEX in AC-AEX.

The SDS-PAGE results also clearly showed the purity improvement along the HIC-CEX-AEX and AC-AEX purification processes, starting with a high number of additional bands indicative of impurities for the sample after TFF. Directly after the first purification step, a clear improvement was detected, and the three protein bands of VP1, VP2, and VP3 could be seen. For AC-AEX, the overall concentration of the rAAV vectors at the end seemed higher than for HIC-CEX-AEX. Some additional bands in the sample containing the full rAAVs were detected between 130 and 250 kDa, which might be the vector DNA.

### 3.2. Different Initial Steps for the Raw Material

**Figure 3:**
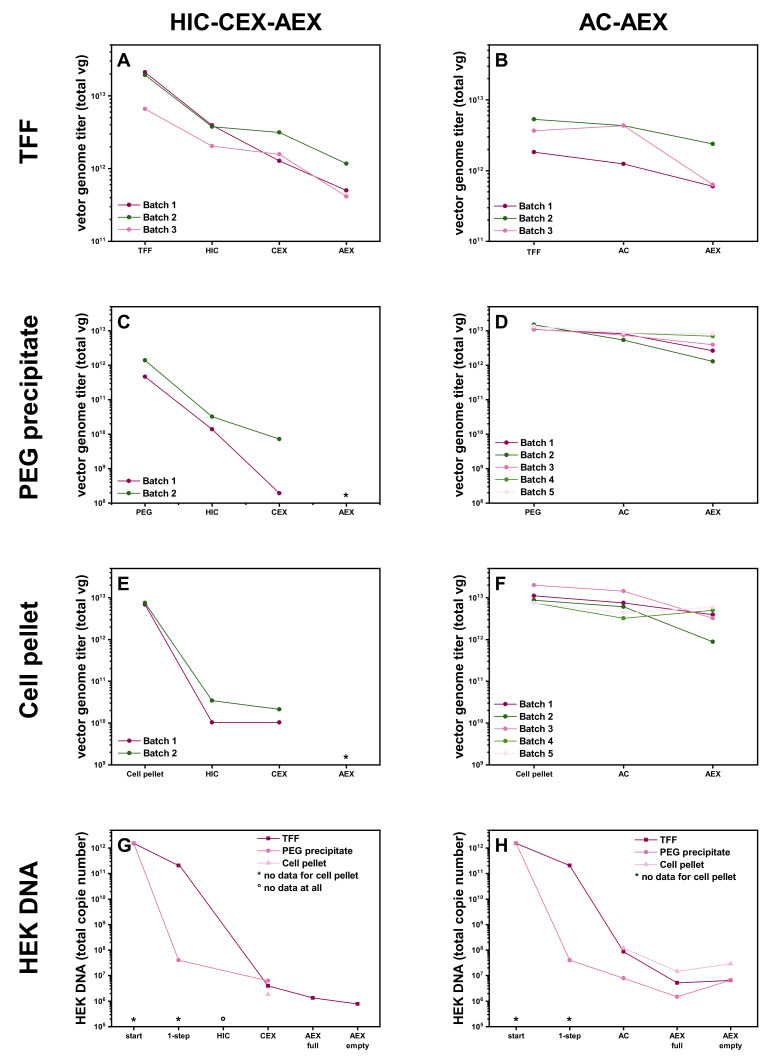
Effect of different harvesting methods on the rAAV2/8 vector production yield and pureness of the human embryonic kidney (HEK) 293T cell DNA**.** (**A**–**F**) Graphs showing the yield (total vector genomes, vg) for the rAAV2/8 vectors obtained with the two strategies, with materials harvested from (**A**,**B**) the cell culture supernatant and cell pellet lysate filtered, concentrated, and buffer-exchanged by tangential flow filtration (TFF) (*n* = 3). (**C**,**D**) Culture supernatant by polyethylene glycol (PEG) precipitation (**C**), followed by HIC-CEX (*n* = 2) and (**D**) followed by AC-AEX (*n* = 5) or (**E**,**F**) cell pellet lysate (**E**) followed by HIC-CEX (*n* = 2) and (**F**) followed by AC-AEX (*n* = 5). (**G**,**H**) Graphs showing the removal efficiency of the strategies for HEK cell DNA at all the purification steps. AC, affinity chromatography; AEX, anion exchange chromatography; CEX, cation exchange chromatography; HIC, hydrophobic interaction chromatography; PEG, polyethylene glycol; rAAV, recombinant adeno-associated virus.

Next, we explored how HIC-CEX-AEX and AC-AEX performed with differently harvested rAAV vector materials. First, we used pooled cell culture supernatants and cell pellet lysates filtered, concentrated, and buffer-exchanged by TFF, which also possibly removed some impurities [32]. Second, we precipitated the rAAV vectors with PEG from the cell culture supernatant only. Third, we pelleted the producer cells and purified the rAAV vectors from the cell pellet lysate. As shown in Figure 3, we were able to consistently purify the rAAV vectors with HIC-CEX-AEX and AC-AEX and from all three starting materials.

According to the vector genome (vg) titers, there was some loss of rAAV vectors during the different steps of the downstream process AC-AEX, ending up with a maximum remaining yield of 44% (4 × 10^12^ vg) after AEX of the initial vg amount (1 × 10^13^ vg). The corresponding values for the different purification steps are summarized in Table 1.

For the HIC-CEX-AEX protocol, we observed a significant drop of the vg titer after the HIC step (Figure 3B,D,F). After the HIC step, we could recover 23% of the rAAVs when starting with material coming from the TFF. In contrast, the recovery was only 3% from the PEG precipitates and only 0.3% when starting with cell pellet lysates. For the samples from the TFF, we performed the subsequent AEX step and could recover 5% of the originally harvested rAAVs in the full fraction. The data are summarized in Table 1. Due to the low yield, we decided not to perform the AEX step with the corresponding samples from the PEG precipitates or the cell pellet lysates.

The determination of the HEK cell DNA showed a clear reduction in HEK cell DNA for the HIC-CEX-AEX and AC-AEX processes. For the starting material of the cell pellet lysate, and for all the samples of the HIC step, it was not possible to determine the amount of HEK cell DNA in the sample because of the matrix effects in the qPCR. We figured out that the first very effective step in the purification process for the supernatant material was PEG precipitation. As expected, the TFF also reduced the amount of DNA and protein impurities but less than the PEG precipitation. In the case of the TFF treatment, the AC, as well as the CEX, were able to reduce the amount of HEK cell DNA. The AEX step further improved the amount of the residual HEK cell DNA in the full rAAV and even more for the empty capsid fraction.

**Figure 4:**
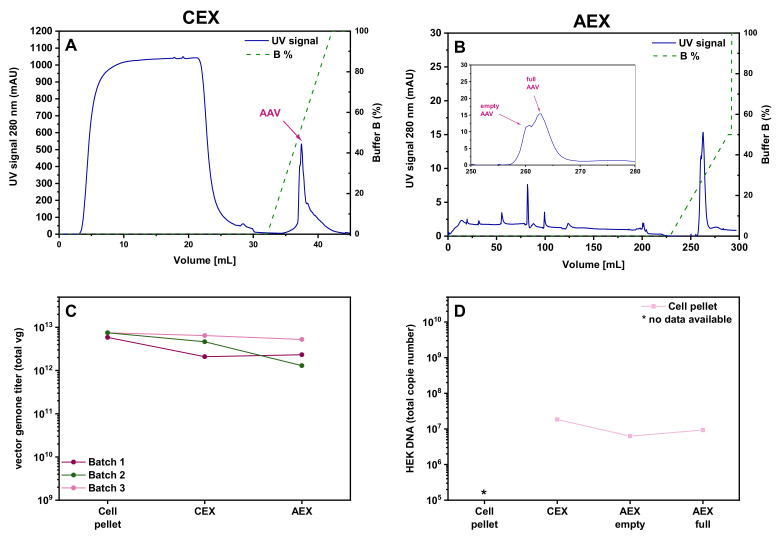
Purification of the rAAV2/8 vectors using the CEX-AEX process. (**A**–**C**) Chromatograms obtained with cell pellet materials in only two steps on the CEX (**A**) and AEX (**B**) columns. (**A**) CEX with a linear buffer B gradient. (**B**) AEX with a linear buffer B gradient. Magnification view of the AEX chromatogram shown in B. Each chromatogram shows the respective UV signal in blue (scale on the left *Y*-axis) and the concentration of buffer B depicted as a dashed green line (scale on the right). (**C**) Graph showing the yield (total vector genomes, vg) for the rAAV2/8 vectors obtained in CEX-AEX with the material harvested by lysis of the cell pellet. The residual HEK cell DNA amount is shown in (**D**). Due to matrix effects in the qPCR, the starting amount cannot be shown here. AAV, recombinant adeno-associated virus; AC, affinity chromatography; AEX, anion exchange chromatography; CEX, cation exchange chromatography.

Given the high loss observed during the HIC step of HIC-CEX-AEX, we decided to test whether it is possible to skip this step and directly load the sample onto the CEX. As shown in the corresponding chromatogram in Figure 4A, omission of the HIC step did not impair proper purification at the CEX column. The same plateau in the UV signal during the sample loading, as in the HIC, was seen in the CEX when the cell pellet lysate material was directly loaded onto the CEX. The amount of rAAVs eluted from the column was 65% for the cell pellet lysate (Table 1). Absolute yield in vg titer for the cell pellet is shown in Figure 4C. Further purification of the sample on the AEX column resulted in a recovery of 59% of the rAAVs from the cell pellet lysate (Figure 4B). Thus, this simplified CEX-AEX process performed better than the original downstream process HIC-CEX-AEX. The residual amount of HEK cell DNA was comparable with the amount in the samples purified by AC-AEX (Figure 4D).

### 3.3. Anion Exchange Chromatography Using a pH Gradient

**Figure 5:**
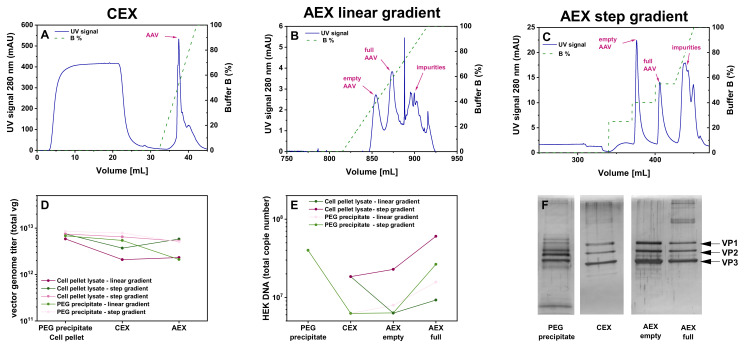
Purification of the rAAV2/8 vectors using CEX-AEX(E/F). (**A**–**C**) Chromatograms obtained with a PEG-precipitated material and a CEX-AEX(E/F) process in only two steps on a CEX (**A**) and a novel CIMmultus PrimaS (AAV) (**B**,**C**) column. (**A**) CEX with a linear buffer B gradient. (**B**) CIMmultus PrimaS (AAV) with a linear buffer B gradient. (**C**) CIMmultus PrimaS (AAV) with a step gradient of 25%, 40%, and 55% buffer B. Each chromatogram shows the respective UV signal in blue (scale on the left *Y*-axis) and the concentration of buffer B depicted as a dashed green line (scale on the right). (**D**) Graph showing the yield (total vector genomes, vg) for the rAAV2/8 vectors obtained in the CEX-AEX(E/F) downstream process with material harvested by PEG precipitation (*n* = 2) from the cell culture supernatant and cell pellet lysate (*n* = 3). (**E**) Residual human embryonic kidney (HEK) cell DNA amount determined by real-time quantitative polymerase chain reaction (qPCR). (**F**) Silver-stained sodium dodecyl sulfate polyacrylamide gel electrophoresis (SDS-PAGE) of the material from each step of the purification CEX-AEX(E/F) downstream process. AEX, anion exchange chromatography; CEX, cation exchange chromatography; PEG, polyethylene glycol; rAAV, recombinant adeno-associated virus; TFF, tangential flow filtration; VP, viral protein.

**Figure A1:**
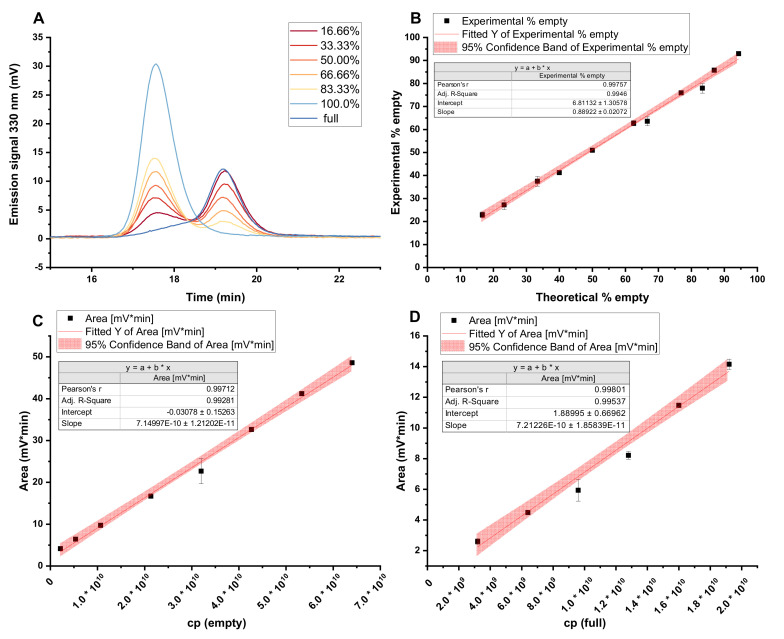
Calibration data of the Protein-Pak Hi Res Q, 5 µm, 4.6 × 100 mm. (**A**) Depiction of the raw data of the chromatograms showing the amount of empty capsids in percent. (**B**) Experimentally determined percentage values of empty capsids plotted against the theoretical percentage. (**C**,**D**) Calibrations of empty capsid (**C**) and full rAAV (**D**) titers.

All tested protocols have so far used the same AEX column (QA column, BIA Separations), which, in principle, allowed for the separation of full rAAVs from empty capsids. However, an optimal (i.e., baseline) separation of the corresponding chromatography peaks could not be achieved. We therefore explored another preparative AEX column (CIMmultus PrimaS (AAV), BIA separations) for full/empty separation. With this column, a linear pH gradient was used for AAV separation, ranging from pH 8.0 to 10.0. The results obtained with this strategy CEX-AEX(E/F) are depicted in Figure 5. A clear separation of the full and empty rAAV peaks could be achieved (Figure 5B). Still, up to 28% of the empty capsids were found in the fractions containing the full rAAVs (Table 2). The residual amount of empty capsids was determined by analytical AEX (Figure A1). We therefore tested a step gradient instead of a linear buffer B gradient. In particular, each step of the B gradient was designed to start at the amount of buffer B that corresponded to the peak maxima previously observed in the linear gradient experiment (Figure 5B).

As shown in Figure 5C, this step gradient protocol resulted in baseline separated peaks for the empty capsids and full rAAVs, respectively. While the separation was improved, as calculated from the analytical AEX, around 19% of the empty capsids were still present in the fractions corresponding to the peak of the full rAAVs. The overall yield of around 70% of the rAAVs from the starting vg amount after CIMmultus PrimaS (AAV) for the CEX-AEX(E/F) process was quite promising (Table 3) and resulted in high genomic titers (Figure 5D).

Next, we assessed the purity profile and found quite a low amount of HEK cell DNA (Figure 5E) and rather low levels of non-VP bands in the SDS-PAGE (Figure 5F). The PEG precipitate as the starting material showed some additional bands around the actual VP1–3 bands, which disappeared after the subsequent purification steps and resulted in marked VP1, VP2, and VP3 bands.

### 3.4. Comparison of Yields of Full rAAVs Harvested from Cell Pellet and Supernatant

**Figure 6:**
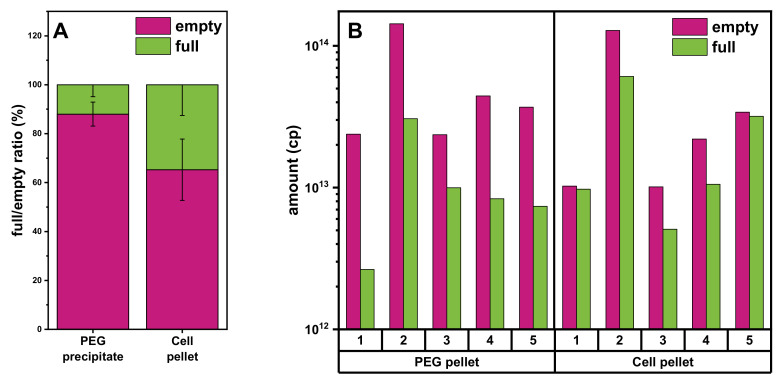
Assessment of the full/empty ratios from the PEG precipitate or cell lysate using analytical AEX chromatography. (**A**) Graph showing the full rAAV (light green) and empty capsid (purple) titer ratio determined using analytical AEX combined with a fluorescence detector. (**B**) Graph depicting the total amount of empty or full capsid values of 5 individual batches obtained from the PEG precipitate or from the cell pellet lysate. PEG, polyethylene glycol.

Comparison of the AEX chromatograms from the experiments with different starting materials revealed a difference in the ratio of empty and full rAAV vectors obtained from the PEG precipitate (Figure 5C) and cell pellet lysate (Figure 4B). We therefore investigated this further. As shown in Figure 6A, the fraction of full over empty rAAVs is higher using cell pellet lysate as the starting material. To analyze this in more detail, we investigated five individual batches and consistently found the ratio of full/empty capsids to be constant in the different conditions and always lower when starting from the PEG precipitate material. Thus, the cell pellet lysate material consistently yielded a higher amount of full rAAV.

### 3.5. Evaluation of AC and CEX Purification Protocols for Four Different Serotypes

**Figure 7:**
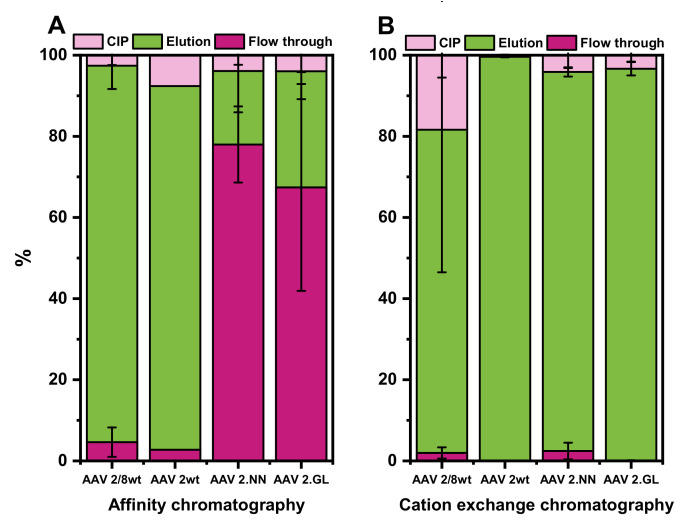
Comparison of AC and CEX for rAAV2/8, rAAV2/2, rAAV2/2.GL, and rAAV2/2.NN. Chromatography with AC (**A**) and CEX (**B**). Purple depicts the flow through and, thus, loss of rAAVs during the loading process onto the column (flowthrough), light green shows the yield of rAAV in the elution, and pink illustrates the loss of rAAVs during the cleaning in place (CIP) process. Minimum, *n* = 3 (except for AC rAAV2/2, *n* = 2)). rAAV, recombinant adeno-associated virus.

So far, the purification methods were evaluated with rAAV2/8 only. We next decided to test the purification of rAAV2/2 and engineered variants thereof only by AC and CEX. The results for “wildtype” rAAV2/2 and engineered rAAV2/2.NN and rAAV2/2.GL are illustrated in Figure 7.

For all serotypes, a minimum of three runs with each column were done. The only exception was for the AC purification of rAAV2/2, for which we performed only two runs. The AC performed very well for rAAV2/8 and rAAV2/2, which could be quantitatively captured on the column. This was not the case for the two engineered rAAV2 variants, which were also bound to the AC column but with a much lower affinity (Figure 7A).

In contrast to the AC, the CEX showed very good results for all four tested types of rAAV, with yields of the remaining rAAVs from the initial vg amount ranging from 93% to 99% in the elution (Figure 7B). rAAV2/8 seemed to bind strongly to the CEX resin, and consequently, up to 20% of the total vector amount was lost, since it could only be eluted from the column during the cleaning in place mode.

## 4. Discussion

Here, we explored different liquid chromatography-based methods for the purification of rAAV material harvested from the cell culture supernatant or from cell pellet lysate. The main findings are summarized in Table 4. For the initial evaluation, we opted to work with rAAVs pseudotyped with the broadly used and easy-to-produce AAV8 serotype.

The HIC-CEX-AEX process included three subsequent chromatographic steps on the HIC, CEX, and AEX columns and consistently produced rAAV2/8 materials at high yields with good purity from the cell culture supernatant, which was initially buffer-exchanged via the TFF. However, there was substantial loss of the rAAV vector when using a cell culture supernatant PEG precipitate or cell pellet lysate (Table 1). A closer inspection of the yield after the different chromatography steps showed that the loss of material mainly happened during the HIC step. The exact reasons for this observation remain unclear. It is tempting to speculate that the initial TFF step helped remove the impurities that negatively impacted the HIC step. Thus, if not combined with an initial TFF step, the HIC-CEX-AEX process resulted in a low yield at the end of the purification process and had to be changed. Indeed, the removal of this initial HIC step in the CEX-AEX process resulted in increased yields from both starting materials, which performed less efficiently in the HIC-CEX-AEX process. Importantly, the CEX column tested in this study showed promising results for all the tested (naturally occurring and engineered) rAAVs. Only for rAAV2/8, a substantial amount of rAAVs remained on the column and, eventually, were lost, since they could only be eluted in the cleaning step.

Further improvements in the procedure, e.g., by adjusting the buffer pH from 4.0 to pH 4.5 or pH 5.0 to reduce binding to the column, might help to increase the fraction of rAAV2/8 that can be recovered in the elution part of the chromatography. It should be noted that longer times at a low pH could damage the capsid, as shown in a recent study [33]. Thus, increasing the pH to optimize binding to the CEX column could also be beneficial for rAAV stability but was beyond the scope of the current work.

In the AC-AEX process, we explored the POROS AAVx affinity column as an alternative to the CEX. In a recent study [29], it was demonstrated that the AAVx column has a high static-binding recovery (>95%) for a broad range of natural and synthetic serotypes. For rAAVs pseudotyped with the naturally occurring AAV serotypes AAV8 or AAV2, we could confirm this observation and achieved a similar recovery for both of them. However, for purification of the two recently described engineered AAV2 variants [22], an affinity to and, thus, recovery from the AC column was substantially reduced, and a high amount of rAAVs was lost in the flowthrough fraction. These AAV2 variants termed AAV2.GL and AAV2.NN carry peptide insertions in the surface-exposed hypervariable loop IV of the AAV capsid, which seems to impact the ability of this capsid to efficiently bind to the affinity matrix. While the exact composition of the affinity matrix was not disclosed by the manufacturer of the AC column, it seems like structural or sequence epitopes made up by the hypervariable loop IV do substantially contribute to the binding affinity.

In addition to the desired vector genome-loaded “full” rAAVs, all rAAV production methods yield varying amounts of genome-free “capsid-only” empty particles as byproducts. Therefore, the HIC-CEX-AEX and AC-AEX processes included a final AEX step, in order to separate the full rAAVs from the empty capsids. The packaging of DNA is thought to induce small changes in the capsid structure and changes in the overall charge of the particles. Indeed, the IEP for empty capsids was reported to be around 6.3 and, for full rAAVs, around 5.9 [13,34,35,36]. In combination with the running buffers with a pH higher than the IEP, this can be exploited for the separation of these two species by AEX chromatography.

Initially, we used the CIMmultus QA column for AEX chromatography in the HIC-CEX-AEX and AC-AEX downstream processes. This column did not achieve a baseline separation of the full and empty peaks but still resulted in an enrichment of the full rAAV. Subsequently, we tested the CIMmultus PrimaS (AAV) column and found a better separation of the full and empty chromatography peaks. In particular, when we applied a step protocol for buffer B, we achieved an almost baseline separation of the full rAAV and the empty capsid peaks. Other studies have also achieved full/empty separation for different AAV serotypes by AEX [8,24,34,37,38,39,40]. For determination of the degree of separation of full rAAVs from empty capsids, we used analytical AEX. To confirm the analytical AEX results, we used SV-AUC as the orthogonal method. Although the SV-AUC analysis at 230 nm provided more sensitivity compared to 260 and 280 nm, only the intermediate and highly concentrated rAAV preparations with absorbance in excess of 0.15 OD units were compared. The AUC consistently worked with the empty capsid fractions and confirmed a very high purity of 99% (compared to 100%, as determined by analytical AEX). The value of the empty capsids that coeluted in the full rAAV fraction was quantified as 17% by SV-AUC, whereas AEX determined the value slightly lower at 12% being empty capsids. The SV-AUC measurements on the full and mixed rAAV fractions also revealed levels of partially filled and aggregated rAAVs, which was not observable by AEX.

Apart from the empty capsids, other impurities are also of relevance. In this regard, we found that rAAV material from the cell pellet lysate ended up having slightly higher amounts of HEK cell DNA compared to rAAV harvested from the cell culture supernatant, either by PEG precipitation or by TFF. The initial step of the buffer exchange via TFF directly removes some process-related impurities, but PEG precipitation was even more effective in removing process-related impurities. However, as seen in the silver-stained SDS-PAGE, both steps did not fully remove the impurities and still resulted in additional bands. Better purification was achieved after HIC, CEX, or AC. An additional AEX step not only helped removing the empty AAV capsids but also further lowered the amount of HEK cell DNA. Interestingly, the AEX step removed the HEK cell DNA from the empty capsid fractions even more efficiently than from the full rAAV fractions. The exact reason for this remains unclear, but it might be related to the fact that full rAAV elutes later in the gradient of the AEX column. Since DNA binds strongly to the AEX column, DNA contaminations elute late in the gradient [6], which coincides more with the elution of the full rAAVs.

Another point to consider when working with the different starting material are potential differences in the post-translational modification (PTM) pattern of the AAV capsid harvested, e.g., from the supernatant or the cell pellet. Such differences might impact on the production process or the properties of the product. Indeed, various PTMs of the AAV capsid have been identified [41,42], and the potential effects on the rAAV vectors function discussed [43]. Whether and how the PTM pattern impacts the chromatographic purification of rAAV needs to be clarified in future studies.

So far, the gold standard for the purification of rAAV are gradient ultracentrifugation-based protocols using cesium chloride [44] or iodixanol gradients [45]. In addition to the removal of impurities, such protocols, in particular when performed with two (or more) subsequent ultracentrifugation steps, are able to efficiently separate full rAAV vectors from empty AAV capsids. The drawbacks of the ultracentrifugation-based protocols are poor scalability and its dependence on the operator, which can lead to high batch-to-batch variability. Therefore, the use of ultracentrifugation-based processes for the large-scale commercial production of rAAVs is challenging. Nevertheless, these methods are frequently used in basic research applications and preclinical in vivo studies [46] and can be considered with some optimizations for the GMP processes for indications of the local application of a vector that do not require a large scale for the manufacturing of high amounts of rAAV vectors.

Other than gradient ultracentrifugation-based protocols, all the chromatography-based steps we tested are readily scalable. AC is highly selective and recovers for rAAV2/8 60–80% of the initial vg amount, but AC does not discriminate between full and empty, and the vg yield depends on the serotype—especially, the vectors produced with engineered AAV capsids are problematic with this type of column. In contrast to AC, the CEX worked with all the tested rAAVs and has comparable yields of around 70–80%. On the other hand, CEX cannot discriminate assembled capsids from protein impurities that show a similar pI and, thus, usually require a subsequent step using an alternative matrix with distinct pH and salt conditions. However, both AC and CEX do not remove the inherent byproduct of empty capsids and, which requires an additional AEX step, which, under optimal conditions, can achieve baseline separation of full and empty rAAVs. Of note, by combining the CEX with an AEX column, the aforementioned issue could be solved and protein impurities removed.

## 5. Conclusions

In conclusion, we presented a comparison of the different chromatographic purification methods, which allow for the efficient removal of the process-related impurities and separation of the full rAAVs from the empty capsids at promising yields. The different protocols showed varying performances for the different serotypes. Our results, thus, can provide guidance for future refinement and the adjustment of chromatographic protocols to a specific type of capsid. Depending on the AAV serotype, a combination of CEX and AEX or AC and AEX is recommend. Full and empty rAAV separation worked particularly well and achieved a high level of purification for the commonly used AAV8 serotype. Our findings hold promise for future translational projects that require highly purified and full particle-enriched rAAV preparations.

## Figures and Tables

**Table 1:** Remaining percentage of the original vg amount of rAAVs after the distinct process steps.

Purification Strategy	Purification Step	TFF (%)	SD (%)	PEG Precipitate (%)	SD (%)	Cell Pellet Lysate (%)	SD (%)
HIC-CEX-AEX	HIC	23	6	3	-	0.3	-
	CEX	15	7	0.3	-	0.2	-
	AEX	5	2	ND	ND	ND	ND
CEX-AEX	CEX	ND	ND	ND	ND	65	13
	AEX	ND	ND	ND	ND	59	24
AC-AEX	AC	89	21	69	24	59	12
	AEX	32	11	44	21	37	23

AC, affinity chromatography; AEX, anion exchange chromatography; CEX, cation exchange chromatography; HIC, hydrophobic interaction chromatography; ND, not done; PEG, polyethylene glycol; rAAV, recombinant adeno-associated virus; SD, standard deviation; TFF, tangential flow filtration.

**Table 2:** Amount of empty capsids in the fractions containing full rAAVs determined by analytical AEX.

Purification Strategy	Number of Preparations	Residual Amount of Empty Capsids in Full rAAV Fractions (%)	
		Entire Peak	Peak Maximum
CEX-AEX(E/F) linear	1	27	21
	2	31	23
CEX-AEX(E/F) steps	1	17	8
	2	17	12
	3	20	9
AC-AEX(E/F) linear	1	23	15
	2	40	28
AC-AEX(E/F) steps	1	20	13

AC, affinity chromatography; AEX, anion exchange chromatography; CEX, cation exchange chromatography; rAAV, recombinant adeno-associated virus.

**Table 3:** Remaining percentage of the original vg amount of rAAV after each different purification step.

Purification Strategy	Number of Preparations	Purification Step	Yield (%)
CEX-AEX(E/F) linear	1: Cell pellet	CEX	64
		AEX	70
	2: PEG pellet	CEX	80
		AEX	31
CEX-AEX(E/F) steps	1: Cell pellet	CEX	86
		AEX	70
	2: Cell pellet	CEX	55
		AEX	78
	3: PEG pellet	CEX	92
		AEX	63

AEX, anion exchange chromatography; CEX, cation exchange chromatography; PEG, polyethylene glycol.

**Table 4:** Comparison of the ultracentrifugation based und liquid chromatography-based purification of the rAAVs.

Purification Strategy	Yield for AAV2/8	Working Time (Hours)	Scalability	GMP Readiness	Full/Empty Separation	Remaining Impurities
ultracentrifugation	good	8–10	poor	moderate	after 2 runs good	iodixanol/cesium chloride
AC	good to very good	2–3	good	good	poor	DNA
CEX	good to very good	0.5–1	good	good	poor	proteins, DNA
AEX	good	2–3	good	good	good	small amounts of DNA

AAV, adeno-associated virus; AC, affinity chromatography; AEX, anion exchange chromatography; CEX, cation exchange chromatography; GMP, good manufacturing practice.

## Data Availability

Data are contained within the article.
